# Targeting FTSJ1 to Restore TP53 Function in Nonsense Mutation-Driven Cancer: Computational and Experimental Evaluation of 1,2,4-Triazole Derivatives

**DOI:** 10.3390/molecules31203709

**Published:** 2026-10-16

**Authors:** Muhammad Sohaib Roomi, Giulia Culletta, Vincenzo Lombardo, Emanuele Vitale, Davide Ricci, Riccardo Varrica, Ignazio Fiduccia, Andrea Pace, Laura Lentini, Ivana Pibiri, Marco Tutone

**Affiliations:** Department of Biological, Chemical and Pharmaceutical Sciences and Technologies, University of Palermo, Via Archirafi 11, 90123 Palermo, Italy; muhammadsohaib.roomi@unipa.it (M.S.R.); vincenzo.lombardo12@community.unipa.it (V.L.); emanuele.vitale@unipa.it (E.V.); davide.ricci@unipa.it (D.R.); riccardo.varrica@unipa.it (R.V.); ignazio.fiduccia@unipa.it (I.F.); andrea.pace@unipa.it (A.P.); laura.lentini@unipa.it (L.L.); ivana.pibiri@unipa.it (I.P.)

**Keywords:** TRIDs, FTSJ1, induced fit docking, unbiased and biased molecular dynamics, nonsense mutation, precision medicine

## Abstract

Nonsense mutations introduce premature termination codons (PTCs) into mRNA transcripts, leading to truncated and non-functional proteins, and accounting for approximately 10–15% of genetic disorders. In this context, the induction of translational readthrough by small molecules, known as translational readthrough-inducing drugs (TRIDs), has emerged as a promising therapeutic strategy to restore full-length protein expression. However, the mechanism of action of TRIDs is still under debate, and one emerging target is FTSJ1, a methyltransferase involved in tRNA modification. FTSJ1 regulates translational fidelity and nonsense-mediated mRNA decay, and its inhibition has been proposed to facilitate the restoration of full-length proteins from nonsense-mutated genes. In this study, we investigated the inhibition of FTSJ1 methyltransferase by a novel series of TRIDs, the 1,2,4-triazole derivatives IP14, IP15, IP17, and IP18, using a luciferase assay in HeLa cells transfected with the pFLuc190UGA plasmid, which in our previous studies showed the rescue of p53 expression in H1299 R213X cells with TP53 gene harboring the R213X nonsense mutation. An integrated computational workflow, including induced fit docking, binding pose metadynamics, molecular dynamics simulations, and MM-GBSA analysis, was employed to characterize their interaction within the FTSJ1 catalytic pocket. The computational results revealed stable binding modes and favorable energetic profiles, supporting the hypothesis that inhibition of FTSJ1 may contribute to their biological activity. Consistent with these findings, in vitro assays supported their translational readthrough activity, suggesting a possible mechanism of action.

## 1. Introduction

Nonsense mutations introduce premature termination codons (PTCs) within coding sequences [1], leading to truncated, non-functional proteins and frequently triggering nonsense-mediated mRNA decay (NMD), a surveillance pathway that further reduces transcript abundance [2,3,4]. These mutations account for approximately 10–15% of inherited diseases and are observed in a significant fraction of tumor suppressor gene mutations, including TP53, which is altered in more than half of human cancers [5]. In detail, PTCs occur when a point mutation turns an amino-acid expressing codon into a stop codon (UAA, UAG, or UGA) located upstream of the natural termination codon (NTC) of the gene, resulting in early termination of translation and loss of protein function [6]. In the case of a nonsense mutation in the gene coding for p53, this leads to the lack of production of a key regulator of DNA damage response, cell-cycle control, and apoptosis, thereby favoring tumor progression [3,5,6,7].

Therefore, in the case of a p53-expressing gene bearing a nonsense mutation, restoring the production of a full-length p53 protein through a translational readthrough (TR)-based therapy has emerged as a particularly attractive approach against cancer development. TR enables ribosomes to bypass PTCs through the incorporation of near-cognate tRNAs, allowing the synthesis of full-length proteins while not involving NTC and preserving endogenous gene regulation [8,9].

Molecules capable of pharmacologically inducing TR are referred to as TR-inducing drugs (TRIDs) [8].

Early TRIDs, such as aminoglycosides, demonstrated proof-of-concept efficacy but are limited by toxicity and poor selectivity [10,11]. Ataluren was the only TRID ever to receive market approval. This non-aminoglycosidic compound was granted conditional approval by the European Medicines Agency (EMA) for Duchenne muscular dystrophy (DMD); however, this approval was recently withdrawn owing to an unfavorable risk–benefit profile, leaving a significant gap in treatment for nonsense mutation-related diseases [12]. Consequently, TRIDs represent a major unmet therapeutic need, and their development has become a central focus in the field.

To this end, pharmacophore-based strategies described in previous studies enabled the identification of novel TRID chemotypes, thereby expanding the chemical diversity of readthrough-inducing molecules and highlighting the importance of integrating computer-aided drug design (CADD) into this field [13,14]. These approaches have also led to significant advances in the development of new oxadiazole-based TRIDs, which promote selective PTC readthrough and exhibit improved safety and efficacy relative to Ataluren in both in vitro and in vivo models [15,16,17]. In addition, these approaches have also been successfully applied in different nonsense mutation-driven contexts, including primary immune deficiency disorders (PIDDs) [18,19]. Together, computational and experimental studies demonstrated that rational scaffold optimization can enhance readthrough efficiency. In this context, although the mechanism of action of aminoglycosides is relatively well established, that of Ataluren has long been debated [20]. Using a reconstituted cell-free translation system, Cooperman and co-workers demonstrated that Ataluren promotes readthrough by selectively inhibiting release factor-dependent termination, through a competitive inhibition of the eRF1-eRF3 complex at ribosomal level, rather than enhancing near-cognate tRNA incorporation [21]. However, increasing evidence suggests that additional cellular factors are involved in RNA processing and translational fidelity and may contribute to readthrough modulation, advancing the hypothesis of a multitarget mechanism.

In this regard, the RNA methyltransferase FTSJ1 [22,23] has recently emerged as a compelling and largely unexplored target. FTSJ1 catalyzes 2′-O-methylation of specific tRNA nucleotides, including modification at the wobble position (Cm34) of tRNA^Trp^, a process essential for maintaining translational accuracy and proper codon–anticodon pairing [24,25,26].

Strikingly, recent hypotheses propose that inhibition of FTSJ1 may reduce translational stringency, thereby facilitating the incorporation of near-cognate tRNAs at PTC sites and enhancing readthrough efficiency. This mechanistic framework introduces a paradigm shift in TRID research, suggesting that modulation of RNA-modifying enzymes, rather than direct ribosomal targeting alone, may represent a viable strategy to potentiate nonsense suppression. Supporting this view, compounds such as 2,6-diaminopurine (DAP) and selected non-aminoglycosidic TRIDs have been reported to promote readthrough via mechanisms consistent with FTSJ1 inhibition [2,9,27]. However, the direct involvement of FTSJ1 in TRID activity remains largely unexplored, and the identification of selective small-molecule inhibitors is still in its infancy. In a recently published paper, our research group discovered four 1H-1,2,4-triazole derivatives capable of restoring full-length p53 protein in H1299 lung adenocarcinoma cells harboring the R213X nonsense mutation without observable toxicity [3].

In particular, all four compounds restored p53 expression with greater efficacy than Ataluren, reaching levels comparable to those achieved with G418, an aminoglycoside antibiotic used as a positive control, while being effective at markedly lower concentrations. Moreover, treatment with the 1H-1,2,4-triazole derivatives restored the p53 response to stress and its transcriptional activity, as evidenced by increased GADD45A expression. Notably, IP17 and IP18 induced a more pronounced increase in p53 expression following exposure to stress stimuli [3].

Here, we investigate the hypothesis that these compounds could exert their biological activity through the modulation of FTSJ1 as one of the mechanisms of action. To this end, we combined an integrated computational workflow, including induced fit docking (IFD), binding pose metadynamics (BPMD), molecular dynamics simulations, and MM-GBSA analysis, with in vitro validation assays. By investigating the potential interaction between 1,2,4-triazole TRIDs (Figure 1) and FTSJ1, this study aims to assess the possible involvement of FTSJ1 in translational readthrough modulation and to provide mechanistic insights that may support the rational design of next-generation TRIDs with improved efficacy and mechanistic specificity.

## 2. Results and Discussion

### 2.1. Induced Fit Docking (IFD)

A previous study has demonstrated that the novel molecule DAP effectively promotes readthrough of premature UGA stop codons in TP53 through its interaction with FTSJ1. This finding highlights a promising therapeutic strategy for the treatment of nonsense mutation-associated diseases and establishes FTSJ1 as a druggable target for restoring functional protein expression [2]. Given the crucial role of FTSJ1 in modulating translational fidelity, a detailed understanding of FTSJ1’s structural features and its catalytic site is essential for the rational design of next-generation readthrough-inducing molecules. We initially considered two crystal structures of the yeast tRNA methyltransferase (PDB ID: 6JP6 and 6JPL), the latter of which contains the co-factor S-adenosyl-L-methionine (SAM) within the active site. We then generated a 3D model of human FTSJ1 via homology modeling by using SWISS-MODEL (https://swissmodel.expasy.org/interactive) (8 September 2026) with 6JP6 as the primary template and incorporated the coordinates of SAM into the final model. To ensure the suitability of the model for subsequent simulations, the resulting structure was subsequently refined using a systematic protocol [27]. This comprehensive refinement yielded a validated structural scaffold that served as the basis for the subsequent computational analyses, including IFD, BPMD, long-timescale MD simulations, and free-energy calculations of the IP-series triazole inhibitors.

Standard rigid docking often fails to adequately account for the flexibility of the catalytic binding pocket because it treats the enzyme as a static receptor. In contrast, IFD incorporates structural flexibility during the protein–ligand binding process [28] and simulates receptor conformational adaptation upon ligand binding, thereby allowing the binding site to more closely accommodate the ligand [29,30]. Accordingly, we employed an IFD protocol to determine the binding orientations of our triazole-based inhibitors. The simulation began by placing each ligand in the binding cavity [28], followed by iterative refinement of the side-chain conformations of the surrounding amino acids, allowing them to move and rotate to achieve an optimal fit with the ligand. This approach accounts for the reciprocal conformational adaptation of the ligand and enzyme during binding. IFD generated a short-list of structurally optimized ligand poses, each representing a plausible binding mode. These poses were then ranked using a scoring function that integrates both shape complementarity and molecular mechanics-based energy terms, yielding a reliable starting geometry for subsequent dynamic analyses and for further validation of the FTSJ1 inhibition hypothesis. Figure 2 illustrates the best-scoring binding poses of the four ligands (IP14, IP15, IP17, and IP18) within the FTSJ1 active site, highlighting their preferred orientations and interactions within the binding pocket.

### 2.2. Binding Pose MetaDynamics (BPMD)

BPMD is used to evaluate the structural stability of the predicted ligand binding modes. BPMD is an enhanced sampling strategy that applies a time-dependent bias to promote the exploration of the ligand conformational space within the binding site. This approach helps distinguish stable binding poses from less favorable ones by monitoring ligand conformational responses under biased conditions [31,32,33]. 

During the simulations, conformational persistence was evaluated by analyzing the movement of each candidate within the binding site using the Collective Variable (CV). Binding modes that showed low root mean square deviation (RMSD) and remained close to their starting geometries under the applied bias were considered more stable, whereas poses showing large RMSD fluctuations indicative of structural instability within the receptor binding site were categorized as less favorable. In addition, the stability of ligand interactions with key binding-site residues was evaluated to determine whether critical contacts were maintained throughout the simulation.

The resulting BPMD stability metrics were assessed using (i) PoseScore, which quantifies the degree of structural drift from the initial docking position, with a sharp increase in PoseScore indicating that the ligand has substantially deviated from its initial binding mode [34]; (ii) PersistenceScore (PersScore), which measures the stability of key polar interactions over the simulation trajectory, specifically focusing on those interactions retained from the original docked structure; and (iii) CompositeScore (CompScore) which was employed as an integrated metric to further assess overall binding stability. CompScore is defined as a linear combination of PoseScore and PersScore, with lower values indicating greater stability of the protein–ligand complex, as described in Equation (1):

CompScore = PoseScore − 5 × PersScore(1)

These metrics were used to rank the IFD-generated poses and assess the binding stability of each ligand by considering the behavior of the corresponding pose under the applied energetic bias, thereby enabling selection of the most reliable protein–ligand complex for subsequent simulations. This filtering step ensured that longer and computationally demanding simulations were initiated from the most stable and structurally plausible binding orientations.

The results in Table 1 showed that among the IP compounds, IP14 and IP17 show the best docking scores, indicating both higher binding and pose stability as compared to DAP and the NV-series 1,2,4-oxadiazole compounds previously reported as TRIDs [27]. Figure 3 illustrates the binding specific intermolecular interactions of the best pose of IP-series compounds within the catalytic pocket of FTSJ1. The proposed triazole derivatives successfully bind to key residues, primarily involving Trp55, Asp116, and Lys156.

Predicted binding orientations and key intermolecular interactions for IP14 (Figure 3a), IP15 (Figure 3b), IP17 (Figure 3c), and IP18 (Figure 3d) illustrate the enhanced network of hydrogen bond interactions and other key interactions. IP14 established a hydrogen bond between its carbonyl group and Arg24, additional hydrogen bonds between its triazole ring and Ser25, Asp116 and Lys156, and aromatic hydrogen bonds between its aromatic ring and Asp47, Cys49 and Asp116. Moreover, pi–pi stacking interaction between the aromatic ring and Trp55 contributes to ligand stabilization. IP15 makes a hydrogen bond interaction between its carbonyl group and Ser25, and another hydrogen bond between its amino group and Asp116. Its triazole ring established pi–cation interaction with Lys28 and Lys156. An additional hydrogen bond exists between the triazole ring and Asp116. Finally, its aromatic ring formed a pi–cation interaction with Arg186. IP17 engaged Arg24 through a hydrogen bond with its carboxyl group. The triazole ring makes two hydrogen bonds with Ser25 and Asp116. Moreover, its aromatic ring establishes aromatic hydrogen bonds with Asp47 and Cys49 to stabilize the ligand within the binding pocket. IP18 forms pi–cation interaction between its aromatic ring and Lys156. In addition, it makes an aromatic hydrogen bond with Asp116, while the triazole ring establishes hydrogen bonds between Ser54, Trp55 and Asp116, stabilizing the ligand within the binding pocket.

While IFD scores are generally comparable across the IP compounds, DAP, and NV-series ligands, the PoseScores of the IP compounds are consistently lower, highlighting their greater stability within the FTSJ1 binding site. IP14 produced one stable pose with a PoseScore of 2.385 Å (Figure 4a). The other values indicate more deviation compared to the other poses, but the stability of the best pose suggests that IP14 may adopt a consistent binding mode once settled. IP15 formed a stable pose, with a PoseScore of 2.676 Å (Figure 4b). The level of deviation of PoseScore for the other poses is lower than that of IP14, suggesting that IP15 achieved a more stable interaction with the protein. IP17 demonstrated the highest level of consistency among all compounds, maintaining five stable poses with almost similar PoseScore values under 2 Å (Figure 4c). This tight clustering of RMSD scores suggests that the ligand binds strongly within the binding site, showing minimal variation across poses. IP18 showed excellent structural stability, with pose 1 exhibiting a low PoseScore of 1.082 Å, having a flat value from the beginning to the end of simulations (Figure 4d). The strong stability of IP18 indicated a highly favorable and persistent interaction with the protein. Considering the weight of PersScore, IP17 and IP18 showed the lowest CompScore, −1.535 and −0.040, respectively, while IP14 and IP15 CompScores were equal to 2.110 and 1.865, respectively, showing similar stability to that of DAP and NVs. Overall, IP17 and IP18 ligands demonstrated promising interactions with FTSJ1 relative to DAP and NV-series compounds, likely due to the substitution of the oxygen ring-atom of the oxadiazole moiety with a NH moiety of the 1-H triazole, which enlarges the possibility of hydrogen bonding for IP14, IP15 and IP17, while this pattern is due to the methoxy group in IP18.

### 2.3. Molecular Dynamics and Thermodynamic Basis for FTSJ1 Inhibition

Molecular dynamics (MD) simulations provide critical insights into the conformational behavior of protein–ligand complexes over time. MD simulations allow the molecular system to be observed within a realistic temporal and spatial framework by modeling the motions of individual atoms under the influence of physical forces [35,36]. In this study, the most stable binding pose identified for each compound by BPMD was used as a starting structure for the corresponding simulations.

We performed three independent replicas, resulting in a total simulation time of 1.5 µs for each complex. To evaluate the behavior of each ligand throughout the simulations, we analyzed the ligand RMSD profiles. This analysis allowed us to track the extent to which each IP compound deviated from its initial binding position over the course of the simulations [37,38], as well as to assess ligand residence within the binding pocket and the overall stability of the protein–ligand complexes. The ligand RMSD profiles reveal distinct stability patterns for the four investigated compounds (Figure 5). Compound IP18 exhibited the most consistent behavior, with all replicas rapidly converging to RMSD values of below ~8 Å, suggesting high structural stability, limited ligand displacement, and retention of the ligand within the binding pocket. Similarly, IP17 showed overall RMSD values of approximately 12–15 Å that were maintained throughout the simulations, indicating a reproducible binding mode and stable accommodation of the ligand within the binding pocket. In contrast, IP14 displayed greater variability among replicas. In all three replicas, the ligand remained within the binding pocket for only a limited number of frames, suggesting a relatively short residence time. These fluctuations suggest the existence of multiple binding conformations and a less reproducible ligand-binding behavior, consistent with the observations from the BPMD analysis. IP15 showed a similar behavior, with substantial RMSD fluctuations and elevated RMSD values persisting over extended periods of the simulations, suggesting conformational rearrangements within or around the binding site. The reference ligand DAP also showed substantial differences in binding stability among the three replicas. In replica 1, DAP maintained a stable binding mode, whereas in replica 2, a stable binding mode was maintained until approximately 250 ns, after which the ligand drifted away from the binding pocket. Although some compounds exhibited relatively short residence times, the IFD and BPMD analyses indicated that they were nevertheless able to establish strong interactions with key binding-site residues.

We present the protein–ligand contacts table to provide a practical overview of both binding strength and specificity. The analysis integrated docking-derived contact maps with MD trajectory frames in which the ligand remained within the binding pocket, allowing us to quantify how interactions within the active site persisted, reorganized, or dissipated over time. We tracked hydrogen bonds, hydrophobic contacts (π–π, π–cation, and non-specific interactions), ionic pairs, and water-mediated bridges, and assessed their occupancy and recurrence across replicas. Reporting MD outcomes on a per-replica basis allowed us to distinguish robust and reproducible contacts from trajectory-specific events. Accordingly, Table 2 integrates the docking-derived interactions with the per-replica MD interaction profiles, providing a coherent, time-resolved view of ligand binding within the active site.

Across the three replicas, IP14 consistently engages Arg24 through hydrogen-bonding interactions, often accompanied by hydrophobic contacts and/or water bridges, indicating a stable anchoring role within the binding pocket. Asp91 is also conserved across all simulations, primarily through hydrogen bonding, highlighting its importance as a persistent polar contact. Lys156 recurs in all replicas with a mixed hydrophobic and hydrogen-bonding profile, suggesting a sustained contribution to ligand stabilization. Additional interactions include Asp75 (replica 1; HB, WB), Ser184 (replica 2; HB, WB), Asp116 (replica 3; HB, WB), and Lys28 in replicas 2 and 3, indicating auxiliary stabilization at the pocket periphery. Overall, the conserved Arg24, Asp91, and Lys156 contacts appear to underpin the shared binding mode of IP14, while the remaining residues provide replica-specific reinforcement of the interaction network.

IP15 exhibits a robust engagement of Asp75, Asp91 and Lys156 across replicas: Asp75 forms hydrogen bonds and water bridges in all three runs (with an added ionic component in replica 3), Asp91 persists as a water-bridged partner that toggles between ionic and hydrophobic character (replicas 2 and 3), while Lys156 interacts predominantly through hydrophobic and/or water-bridged contacts, supporting a recurrent interaction at this basic site. Replica-specific features include Ala51 (WB or hydrophobic/WB) in replicas 1–2, Trp55 hydrophobic contact in replica 3, and distinct glutamine partners Gln134 (replica 1) and Gln87 (replica 2)—providing additional H-bonding capacity. Asp116 appears as a water bridge in replica 3. Taken together, the persistence of Asp75, Asp91, and Lys156 across trajectories defines the core binding pattern, with auxiliary polar contacts varying among replicas.

For IP17, the contact pattern is highly conserved around Arg24, Trp55, Asp116, and Lys156. Arg24 appears in all three replicas, combining hydrogen bonding with ionic and/or water-mediated interactions, while Trp55 maintains hydrophobic contacts throughout, indicating a stable aromatic engagement. Asp116 is repeatedly contacted via a water bridge in every replica, and Lys156 shows ionic and water-bridged interactions (ionic only in replica 3), reinforcing basic-site coordination. Additional residues modulate this core: Arg186 contributes H-bonds, hydrophobic, and water-bridged contacts in replicas 1 and 3 (with an extra HB, WB entry also noted), Trp21 forms H-bond and WB contacts in replica 2, and Glu191 contributes a water bridge in replica 3. The recurrence of Arg24/Trp55/Asp116/Lys156 across all runs supports a stable, reproducible interaction network, with Arg186 serving as a frequent auxiliary anchor. 

IP18 maintains a consistent framework of contacts centered on Arg24, Asp116, and Lys156 across all replicas. Arg24 is present in every run, primarily via hydrogen bonds and water bridges (with added hydrophobic character in replica 3), while Asp116 shows repeat hydrogen-bond/water-bridge participation and Lys156 remains engaged through hydrophobic and/or water-bridged contacts in all replicas. Trp55 forms hydrophobic contacts in replicas 1 and 2, indicating a frequent aromatic stabilization that is absent in replica 3. Gly53 contributes to replicas 1 and 3 (WB in replica 1; HB, WB in replica 3), and Asp75 appears as a water-bridged partner in replicas 1 and 3. Replica-specific contacts include Ala50 hydrophobic in replica 2. Overall, the Arg24–Asp116–Lys156 triad provides a stable backbone for binding, augmented by periodic contributions from Trp55 and Gly53 that likely enhance pose persistence within the pocket.

To provide a biophysical rationale for the observed binding stabilities, we then calculated the binding free energies (ΔG) of the FTSJ1-IP complexes using the Molecular Mechanics-Generalized Born Surface Area (MM-GBSA) based on trajectories from all three MD simulation replicas [39,40,41]. MM-GBSA is a widely applied computational method for approximating the free energy of binding between a protein and a ligand. MM-GBSA estimates binding free energy by combining molecular mechanics contributions, primarily van der Waals and electrostatic interactions, with polar and non-polar solvation contributions. This approach has been widely applied to characterize relative binding affinities and ligand–protein interactions from molecular dynamics trajectories [42]. For each complex, MM-GBSA calculations were performed independently for the three MD replicas, and the mean binding free energy was calculated together with the corresponding standard error (SEM) (Figure 6). IP14 and IP15 demonstrated ΔG similar to that of DAP. In contrast, IP17 and IP18 exhibited higher binding affinities than DAP, indicated by more negative ΔG values. The enhanced stability of IP18 aligns with its sustained binding behavior and persistent hydrogen-bond interactions observed during both BPMD and MD simulations. Thus, these findings complement the in vitro analysis and further support the potential of these compounds as FTSJ1 inhibitors.

### 2.4. In Vitro Luciferase Assays in Hela Cells

To further investigate our in silico model and the possibility that IP compounds induce readthrough by affecting FTSJ1 activity, a luciferase assay was performed at different IP concentrations. These in vitro tests were conducted to evaluate a possible molecular mechanism of readthrough induction exploited by this new class of TRID compounds. Thus, HeLa cells were both transiently transfected alone with a pFLuc190UGA plasmid and co-transfected with a plasmid harboring the FTSJ1 cDNA (kindly donated by the Lejeune group) and treated with IP molecules [2].

In addition, as a positive control, they were also transfected with a pFLucWT vector (positive control). Thus, 24 h post-transfection, the HeLa cells transfected alone with the pFLuc190UGA plasmid (1 µg) and co-transfected with the FTSJ1 cDNA-containing plasmid (2 µg) were treated for 24 h with IP14, IP15, IP17, and IP18 at 6, 12, and 24 µM, and using the Steady-GloMax kit, the luminescence was assessed. The results shown in Figure 7, clearly suggested not only the readthrough activity of the molecules but also their interaction with FTSJ1 to perform their function. Specifically, the increased luminescence observed in pFLuc190UGA HeLa cells treated with IP molecules, compared with untreated cells, demonstrated the readthrough activity of these molecules. Conversely, the reduced luminescence levels observed in the pFLuc190UGA HeLa cells co-transfected with the FTSJ1 plasmid and treated with all IP molecules at different concentrations, presenting an FTSJ1 overexpression, demonstrate an impaired readthrough activity of the IP molecules, suggesting a possible inhibitory activity of IP14, IP15, IP17, and IP18 on FTSJ1 under standard conditions to induce the readthrough. These experimental results are consistent with the computational analysis, which showed that all four IP compounds bind to the FTSJ1 active site. Among them, IP17 and IP18 showed the most favorable computational profiles, with IP17 displaying the lowest CompScore in the BPMD analysis, while both compounds showed more favorable MM-GBSA binding free energies than DAP. These findings provide a structural rationale for the observed effect of FTSJ1 overexpression on IP-induced readthrough and support the involvement of FTSJ1 in the mechanism of action of the IP compounds.

## 3. Materials and Methods

### 3.1. Structure Modeling and Receptor Refinement

The framework of the human tRNA (cytidine(32)/guanosine(34)-2′-O)-methyltransferase (FTSJ1) was downloaded from UniProt: Q9UET6) and modeled within the SWISS-MODEL platform [43,44,45]. The crystal structure of the yeast methyltransferase Trm7 in complex with Trm734 (PDB ID: 6JP6) was selected as the primary template, providing 50.0% sequence identity. The resulting model yielded a Qualitative Model Energy ANalysis (QMEAN) score of 0.75, indicating the overall high structural quality and reliability [46].

Further refinement was performed using the built-in Protein Preparation Wizard in Maestro (Schrödinger New York, 2025-4 ) [47]. This process involved the addition of missing hydrogen atoms, the reconstruction of incomplete side chains and loops, side-chain orientations of asparagine (Asn), glutamine (Gln), and the protonation and tautomeric states of ionizable residues histidine (His), Asp, and Glu, which were assigned a physiological pH (7.20 ± 0.20). Finally, a restrained minimization was performed using the OPLS4 force field [48] to resolve atomic overlaps and optimize geometry while preserving the overall structural stability of the generated model.

### 3.2. Induced Fit Docking and Binding Pose MetaDynamics (BPMD)

Induced fit docking (IFD) is a method for modeling the conformational changes induced by ligand binding developed by Schrödinger [49]. We used this protocol as reported in [50,51,52]. The grid box for the binding site was built considering the residues previously identified in [27]. During the initial docking stage, a van der Waals scaling factor of 0.5 was applied to both receptor and ligand. Prime refinement was performed on side chains within 5 Å of the ligand. For each ligand, up to 20 poses were retained and redocked in Glide XP mode for final scoring.

BPMD is an automated protocol based on metadynamics that enhances sampling efficiency. In these simulations, ligands are encouraged to move within their binding site by an external bias energy function while maintaining their initial pose. This approach has proven effective in distinguishing the correct binding conformation from alternative poses generated through docking or induced fit docking methods [29].

All simulations were carried out following a previously established procedure [50,53,54,55]. After completion, ligand stability throughout the simulation is evaluated using three metrics: PoseScore, Persistence Score (PersScore), and Composite Score (CompScore).

### 3.3. Unbiased Molecular Dynamics Simulations and Binding Thermodynamics

The conformational stability of the FTSJ1/IP complexes was analyzed through MD simulations carried out using the Desmond (Schrodinger 2023-1) [55] and the OPLS4 force field. Each complex was solvated within a cubic box of water molecules (TIP3P) and counter-ions, and with temperature set to 310 K to mimic physiological conditions, and after equilibration, production of MD simulations was carried out for 500 nanoseconds. The system setup and the simulation options were the same as reported in previous manuscripts [50,52]. To ensure flexibility, three independent replicas were performed for each of the four compounds using randomized initial velocity seeds to ensure conformational sampling and reduce bias from the starting configuration. Throughout the simulations, the structural stability of the binding modes was monitored by tracking parameters such as RMSD, residue fluctuations, and the persistence of key interactions, including hydrogen bonds and hydrophobic contacts. Each pre-generated complex was simulated on a single computational node equipped with an NVIDIA GeForce RTX2070 GPU.

To further estimate binding affinity, the MM-GBSA method [42] was incorporated into representative frames (1000 frames) extracted from the MD trajectories. This approach breaks down the overall binding free energy into molecular mechanics and solvation energy contributions, thus providing a balanced compromise between computational cost and energetic insights. The entropic contribution (−TΔS) was not included, as the primary objective was to compare relative binding affinities across the IP series rather than determine absolute free energy values.

### 3.4. TRIDs Synthesis and Preparation

IP14, IP15, IP17, and IP18 were synthesized as previously reported [3] and resuspended in dimethyl sulfoxide (DMSO) (Merck, Rahway, NJ, USA) in different concentration stock solutions for each compound. Thus, a work solution for each compound at 1 mM was prepared to treat the cells by diluting the stock solution in Dulbecco’s Phosphate-Buffered Saline 1X (DPBS 1X) (Corning, NY, USA). A 2% DMSO vehicle control solution was prepared by mixing 20 µL of DMSO with 980 µL of DPBS1X and used at a final DMSO concentration of 0.048% in the control samples.

### 3.5. Cell Culture

HeLa cells were grown in Dulbecco’s Modified Eagle Medium (DMEM) (Corning, NY, USA) supplemented with 1% L-glut (Corning, NY, USA), 1% P + S antibiotics (Corning, NY, USA), and 10% Fetal Bovine Serum (Corning, NY, USA) at standard conditions of 37 °C and 5% CO_2_.

### 3.6. Luminescence Assay

A total of 2.5 × 10^5^ HeLa cells were plated into each well of the 6 MW plate. Twenty-four hours after seeding, a total of 1 µg of pFLuc190UGA plasmid was transfected alone or co-transfected with 2 µg of FTSJ1 plasmid by using Turbofectamine reagent (Thermofisher, Vilnus, Lithuania), according to the kit instructions. As positive control, 1 µg of the pFLuc-WT plasmid was transfected alone. A 24 h treatment on HeLa cells was performed with 6 µm, 12 µm, or 24 µm for each IP molecule, 24 h after transfections, while in the control samples only the highest final DMSO concentration (0.048%) was tested. On the following day, cells were incubated for 5 min with 400 µL/well of the detection mix from the Steady-Glo luciferase kit (Promega, Madison, WI, USA). Cell suspensions (200 µL) were plated in duplicate in 96-well plates, and luciferase activity was quantified using a luminometer. The experiment was performed in two biological replicates, each with two technical duplicates. Data were analyzed in GraphPad Prism 6 using two-way ANOVA. Statistical significance was defined as < 0.05 and is represented in figures as follows: (*p*): one symbol (*) for *p* < 0.05, two symbols (**) for *p* < 0.01, three symbols (***) for *p* < 0.001, four symbols (****) for *p* < 0.0001.

## 4. Conclusions

In this study, we investigated the potential role of FTSJ1 inhibition as a mechanism underlying the translational readthrough activity of a novel series of 1,2,4-triazole derivatives: IP14, IP15, IP17, and IP18, which were previously shown to restore p53 expression in cells harboring the TP53 R213X nonsense mutation. By integrating induced fit docking, binding-pose metadynamics, long-timescale molecular dynamics simulations, MM-GBSA binding free-energy calculations, and in vitro validation assays, we provide converging evidence supporting the ability of these compounds to interact with the catalytic pocket of FTSJ1. Computational analyses indicated that all four compounds can accommodate the FTSJ1 active site and establish interactions with key residues involved in ligand recognition and stabilization. Among the investigated molecules, IP17 and IP18 exhibited the most favorable overall profiles, characterized by stable binding poses, persistent interaction networks across independent molecular dynamics replicas, and more favorable predicted binding free energies than the reference inhibitor DAP. In particular, interactions involving Arg24, Trp55, Asp116, and Lys156 were recurrent across the simulations and emerged as important contributors to ligand stabilization within the catalytic pocket. The enhanced binding stability observed for IP17 and IP18 supports the potential of the 1,2,4-triazole scaffold as a promising structural framework for the development of FTSJ1-targeting translational readthrough-inducing drugs.

The computational findings were further supported by biological experiments. Luciferase-based assays showed that overexpression of FTSJ1 significantly reduced the readthrough activity induced by the IP compounds, providing experimental evidence that their activity is associated with FTSJ1 modulation. These results are consistent with the hypothesis that reduced FTSJ1 activity may decrease translational stringency and facilitate suppression of premature termination codons, thereby promoting the synthesis of full-length proteins from nonsense-mutated transcripts. Although the present findings do not establish direct enzymatic inhibition of FTSJ1, the agreement between the computational binding analyses and the cellular overexpression experiments provides a mechanistically coherent basis for proposing FTSJ1 as a molecular target of this compound series.

Overall, this work strengthens the emerging concept that RNA-modifying enzymes such as FTSJ1 may represent promising therapeutic targets for the treatment of nonsense mutation-related diseases. The identification of IP17 and IP18 as particularly promising FTSJ1 modulators provides a valuable starting point for the structure-guided optimization of next-generation translational readthrough-inducing drugs with improved potency and mechanistic specificity. More broadly, the study demonstrates the value of combining advanced computational approaches with experimental validation to investigate molecular mechanisms and guide the development of precision therapeutic strategies for genetic disorders and cancers driven by nonsense mutations. Future studies, including direct biochemical assessment of FTSJ1 enzymatic activity and structural characterization of ligand–FTSJ1 complexes, will be important to establish the inhibitory mechanism and further validate FTSJ1 as the direct molecular target of these compounds.

## Figures and Tables

**Figure 1:**
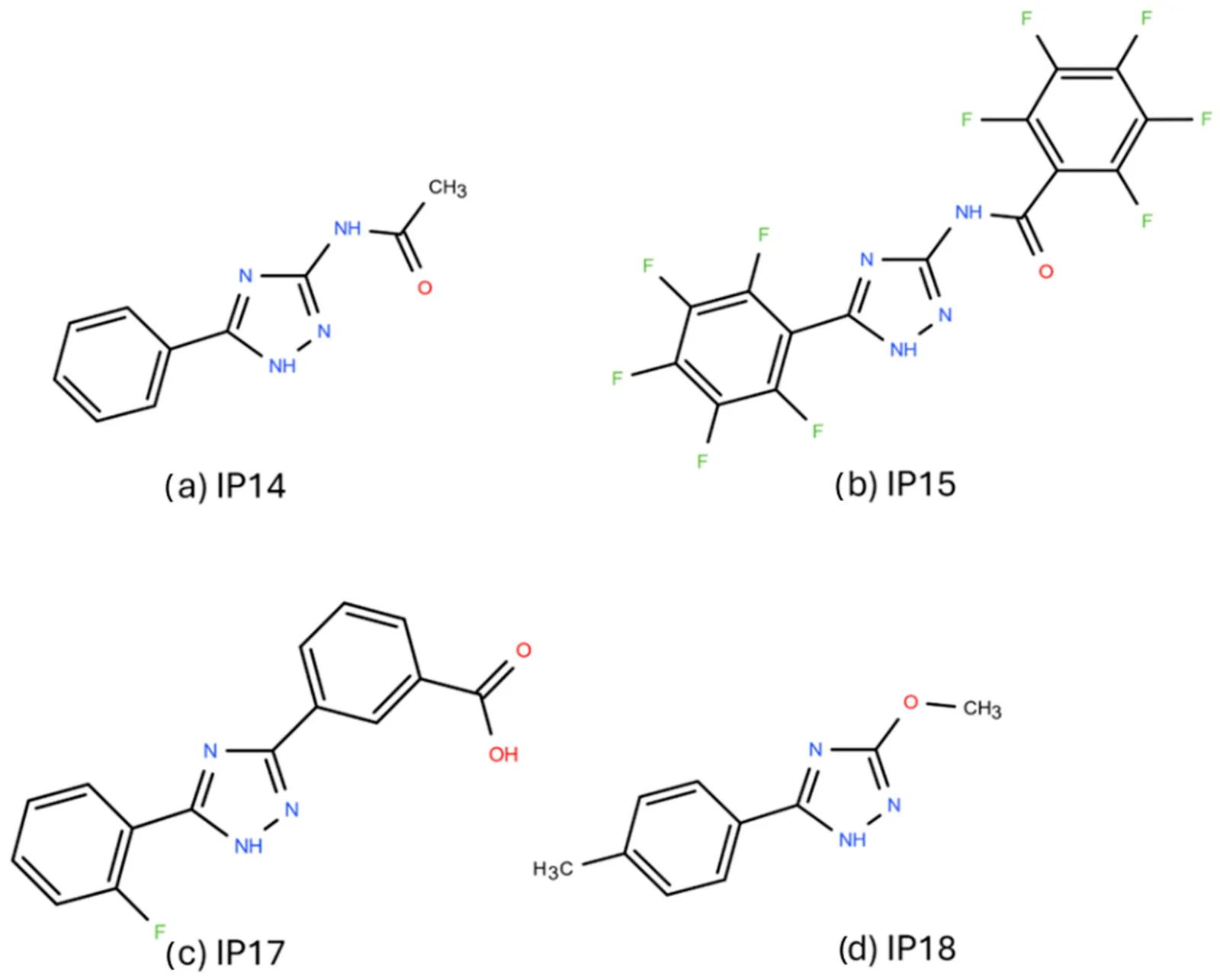
Chemical structures of the proposed triazole-based TRIDs: (**a**) IP14, (**b**) IP15, (**c**) IP17, and (**d**) IP18.

**Figure 2:**
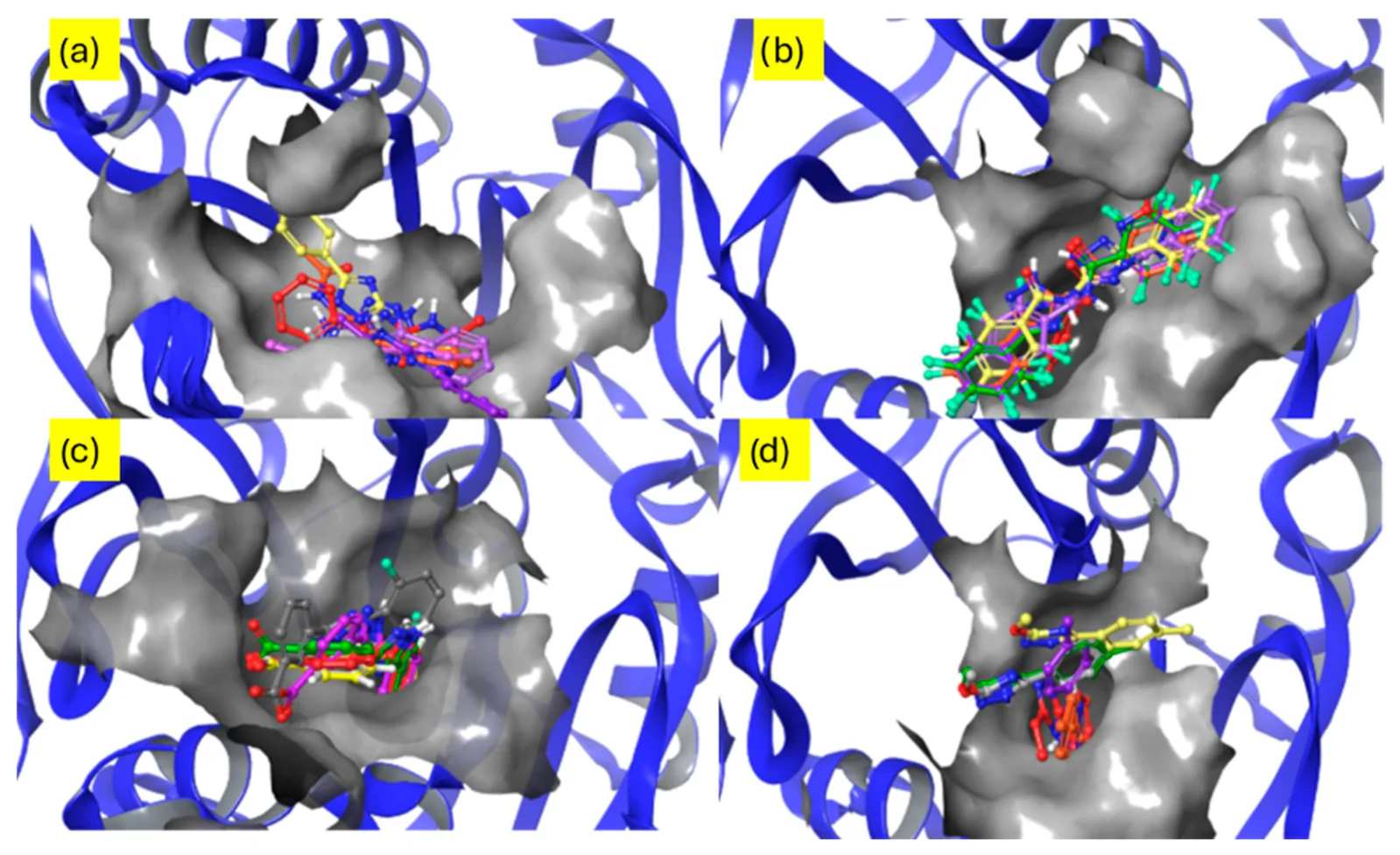
Best-ranked IFD binding poses of (**a**) IP14, (**b**) IP15, (**c**) IP17, and (**d**) IP18 within the FTSJ1 active site, highlighting their spatial arrangement and potential interactions. Each color represents the pose of the ligand. We used the output of IFD as a starting point for the other computational experiments.

**Figure 3:**
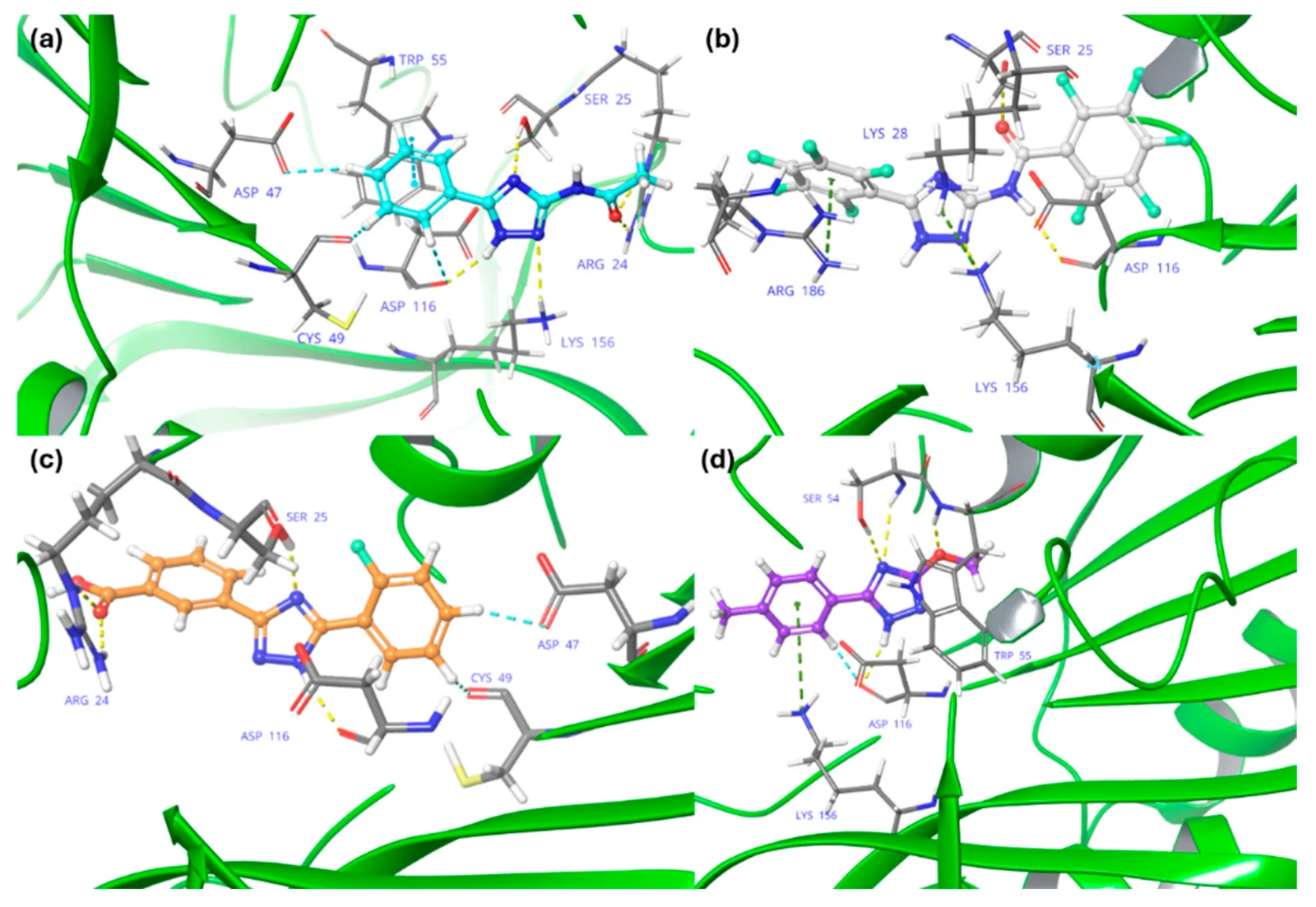
Graphical illustration of the binding modes of IP—series inhibitors within the FTSJ1 catalytic pocket. Ligands are depicted as ball-and-stick models, while critical receptor residues are shown as grey sticks. Yellow-dashed lines: H-bonds; green-dashed lines: pi-cation stacking interactions; light-blue-dashed lines: aromatic H-bonds; blue-dashed lines: pi-pi stacking interactions. (**a**) IP14; (**b**) IP15; (**c**) IP17; (**d**) IP18.

**Figure 4:**
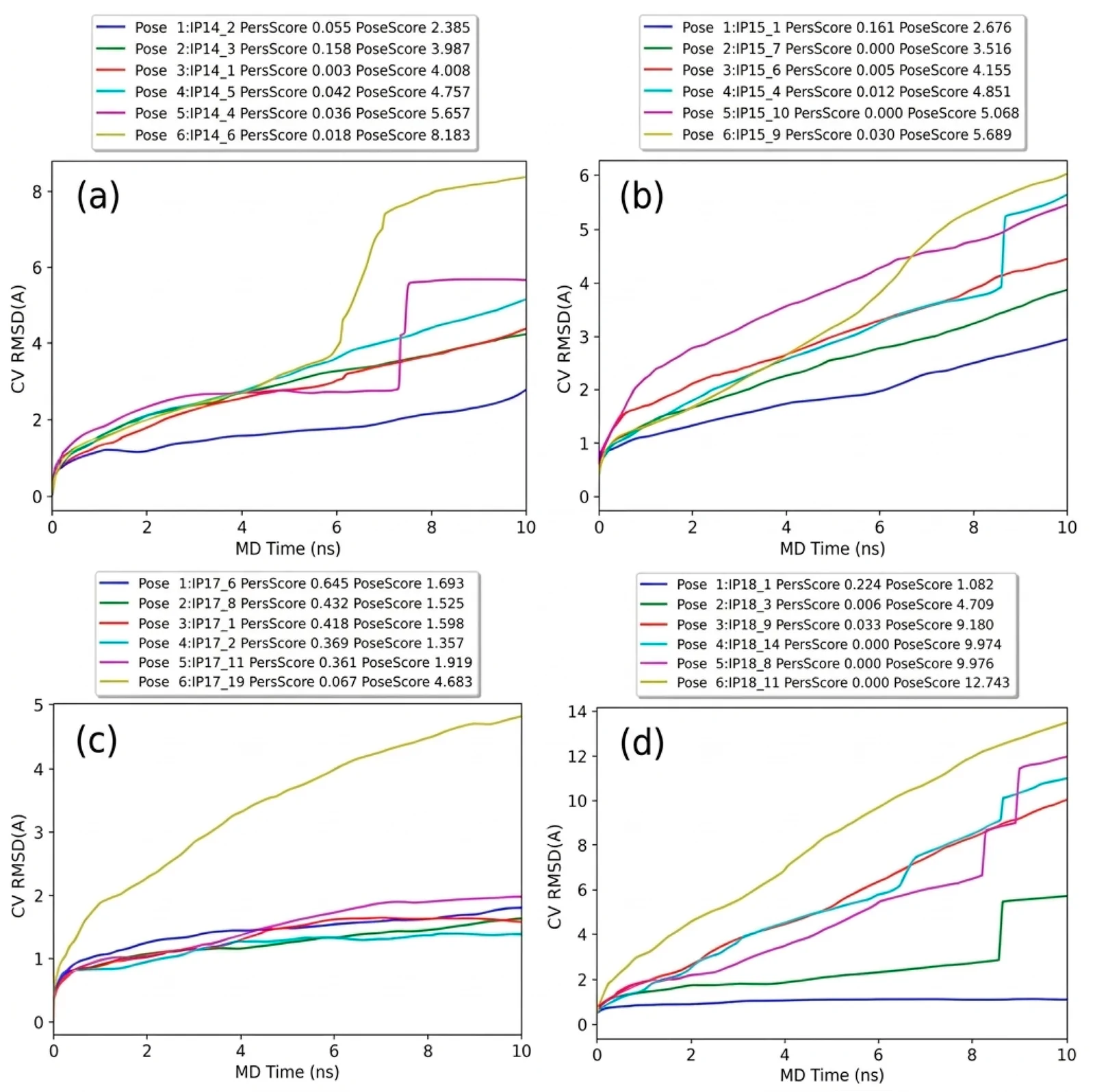
RMSD-based analysis of the predicted binding poses for the four ligands in the FTSJ1 active site: (**a**) IP14; (**b**) IP15; (**c**) IP17; (**d**) IP18.

**Figure 5:**
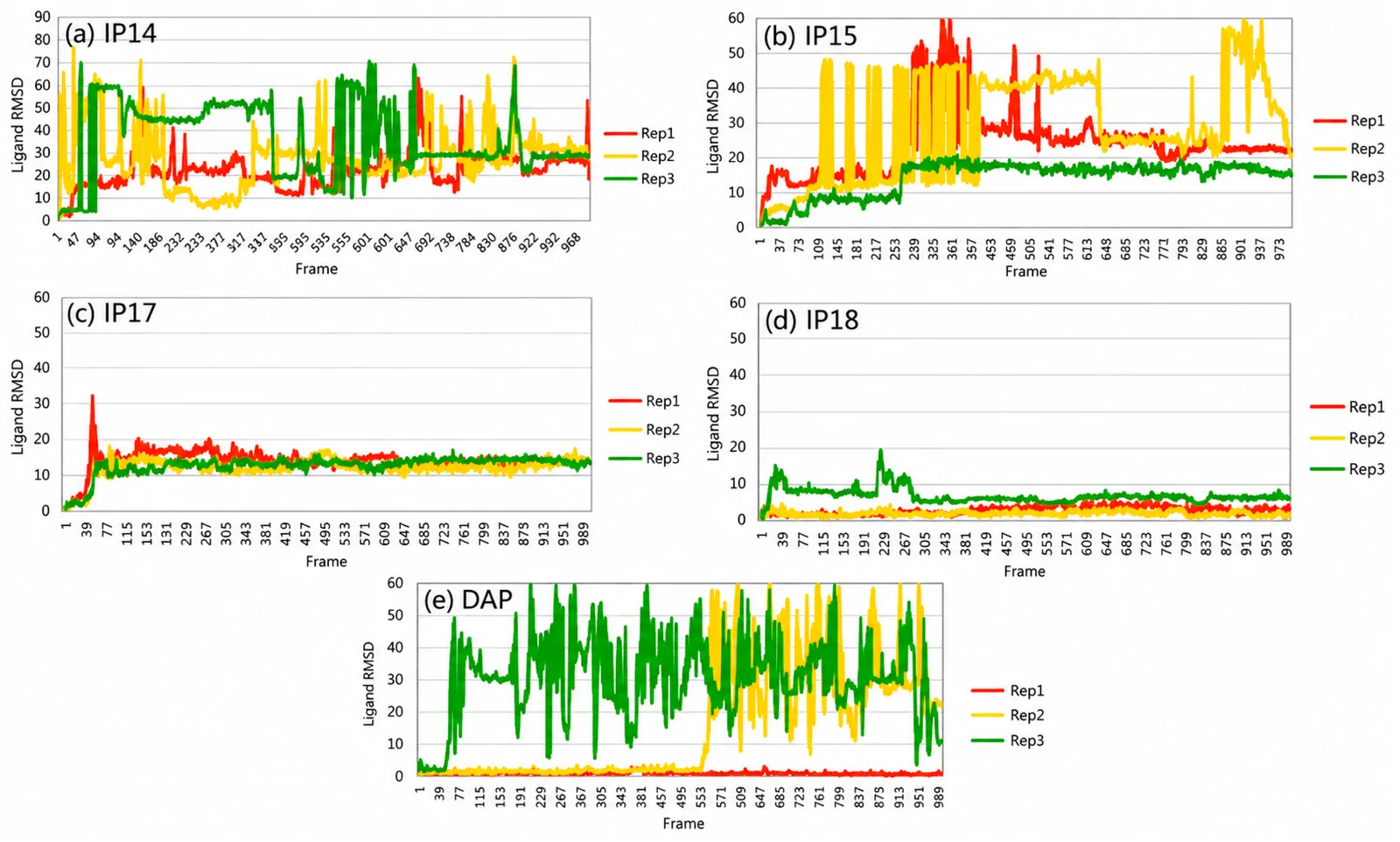
RMSD comparison analysis of FTSJ1 complexes with (**a**) IP14, (**b**) IP15, (**c**) IP17, (**d**) IP18, and (**e**) DAP over three 500 ns MD simulation replicas.

**Figure 6:**
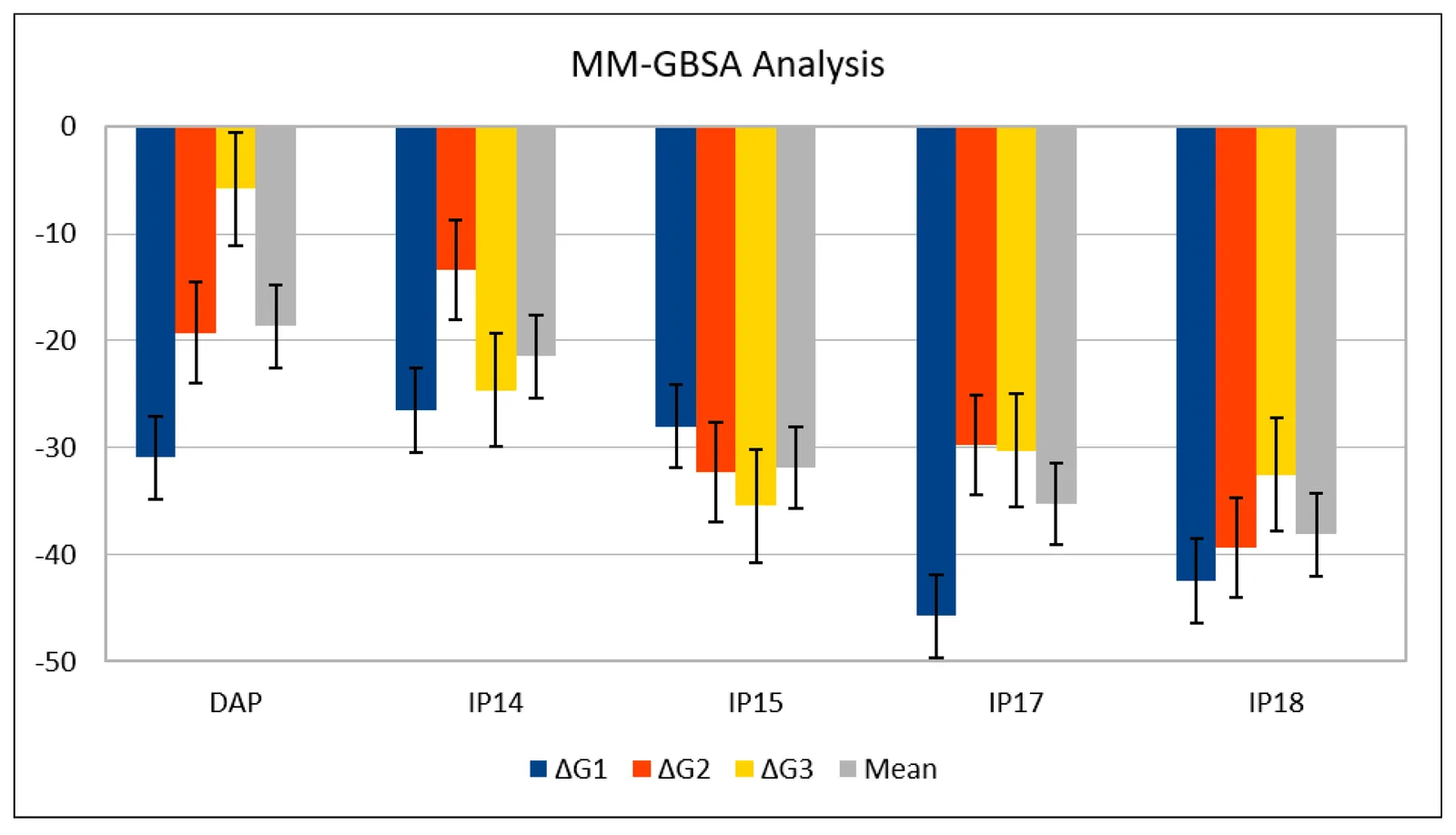
Comparative MM-GBSA binding free energy (ΔG_bind) analysis of the FTSJ1–ligand complexes over 1000 MD simulation frames from three independent replicas. Bars represent the mean ΔG_bind values, and error bars indicate the standard error of the mean (SEM). ΔG_bind values are expressed in kcal/mol.

**Figure 7:**
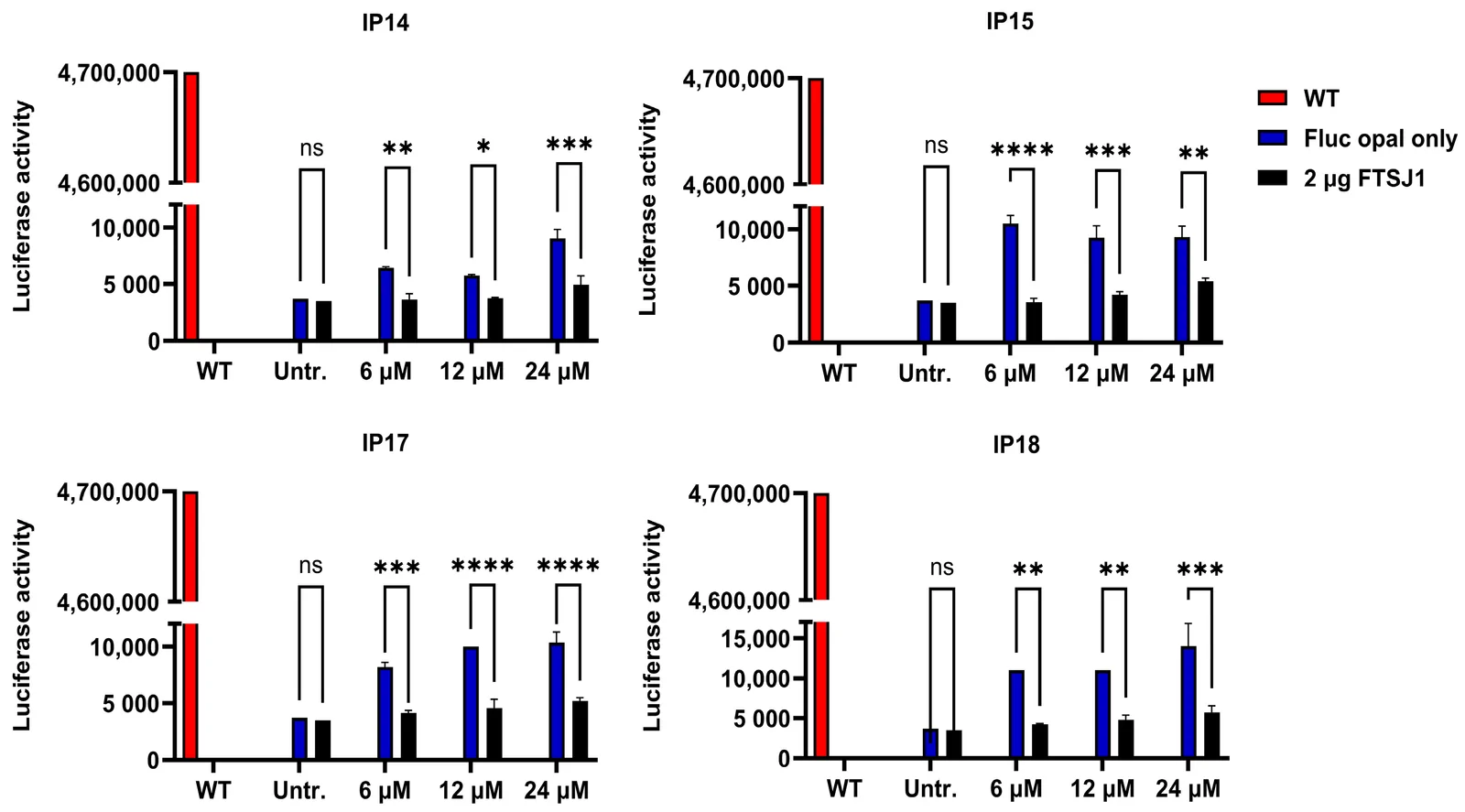
Luciferase assay in HeLa cells: cells were transfected alone with pFLuc190UGA (1 µg) or co-transfected with the FTSJ1 plasmid (2 µg) and treated for 24 h with IP compounds at 6, 12, and 24 µM; of interest, overexpression of FTSJ1 reduces luciferase activity even in the presence of IP treatments. HeLa cells transfected with the FLuc WT plasmid are used as a positive control, and pFLuc190UGA cells as a negative control. Data were analyzed using GraphPad Prism 6. Probability values (*p*): one symbol (*) for *p* < 0.05, two symbols (**) for *p* < 0.01, three symbols (***) for *p* < 0.001, four symbols (****) for *p* < 0.0001.

**Table 1:** Shows the comparison of induced fit docking (IFD), BPMD-derived PoseScores, PersScores, and CompScores between IP, NV, and DAP molecules. The table includes the four IP compounds alongside DAP and the three already proposed TRIDs (NV914, NV848, NV930). Docking Score and IFD Score (in kcal/mol) reflect the predicted binding affinity, while PoseScore (in Å) from BPMD indicates the stability of the ligand within the FTSJ1 binding site, with lower values representing more stable binding poses. PersScore reflects the persistence of key polar interactions over the simulation time, and CompScore integrates PoseScore and PersScore as a combined stability metric, with lower values indicating more stable protein–ligand complexes.

Ligand	Docking Score (kcal/mol)	IFD Score (kcal/mol)	PoseScore (Å)	PersScore	CompScore
IP14	−6.86	−469.01	2.385	0.055	2.110
IP15	−6.20	−468.90	2.67	0.161	1.865
IP17	−6.77	−468.74	1.69	0.645	−1.535
IP18	−6.45	−468.30	1.08	0.224	−0.040
DAP	−6.61	−426.53	3.76	0.255	2.485
NV914	−5.37	−429.84	3.29	0.223	2.175
NV848	−5.46	−427.03	3.06	0.159	2.265
NV930	−6.23	−426.06	4.37	0.322	2.76

**Table 2:** Target–ligand interactions were observed during the MD simulations. Abbreviations: Cpd = compounds, Int*. = interactions, HB = H-bonds, Hphobic = hydrophobic interactions, WB = water bridges. Interactions that are identified in all three replicas are reported in bold.

Cpd	Replica 1		Replica 2		Replica 3	
	Residue	Int*	Residue	Int*	Residue	Int*
IP14						
	**Arg24**	HB, WB	**Arg24**	HB, Hphobic	**Arg24**	HB, Hphobic, WB
	Asp75	HB, WB	Lys28	HB, Hphobic	Lys28	HB, WB
	**Asp91**	HB	**Asp91**	HB, WB	**Asp91**	HB
	**Lys156**	Hphobic, HB	**Lys156**	Hphobic	**Asp116**	HB, WB
			Ser184	HB, WB	**Lys156**	HB, Hphobic, WB
IP15						
	Ala51	WB	Ala51	Hphobic, WB	Trp55	Hphobic
	**Asp75**	HB, WB	**Asp75**	HB, WB	**Asp75**	HB, ionic, WB
	**Asp91**	WB	**Asp91**	ionic, WB	**Asp91**	Hphobic, WB
	Gln134	HB, WB	Gln87	HB, WB	Asp116	WB
	**Lys156**	Hphobic, WB	**Lys156**	Hphobic, WB	**Lys156**	WB
IP17						
			Trp21	HB, WB		
	**Arg24**	HB, ionic, WB	**Arg24**	HB, WB	**Arg24**	HB, ionic, WB
	**Trp55**	Hphobic	**Trp55**	Hphobic	**Trp55**	Hphobic
	**Asp116**	WB	**Asp116**	WB	**Asp116**	WB
	**Lys156**	ionic, WB	**Lys156**	ionic, WB	**Lys156**	ionic
					Glu191	WB
IP18						
	**Arg24**	HB, WB	**Arg24**	HB, WB	**Arg24**	Hphobic, WB
	Gly53	WB	Ala50	Hphobic	Gly53	HB, WB
	Trp55	Hphobic	Trp55	Hphobic	Asp75	WB
	Asp75	WB				
	**Asp116**	HB, WB	**Asp116**	HB, WB	**Asp116**	WB
	**Lys156**	Hphobic, WB	**Lys156**	WB	**Lys156**	Hphobic, WB

## Data Availability

The data presented in this study are available from the corresponding authors upon reasonable request.
